# A Simple, Accurate and Cost-Effective Capillary Electrophoresis Test with Computational Methods to Aid in Universal Microsatellite Instability Testing

**DOI:** 10.3390/cells10061401

**Published:** 2021-06-05

**Authors:** James Wei Tatt Toh, Puneet Singh, Venkata A. A. S. K. Tangirala, Alex Limmer, Kevin J. Spring

**Affiliations:** 1 Department of Colorectal Surgery, Westmead Hospital, Westmead, NSW 2145, Australia; alex.limmer@gmail.com; 2 Westmead Clinical School, The University of Sydney, Westmead, NSW 2145, Australia; 3 Ingham Institute for Applied Medical Research, Liverpool, NSW 2145, Australia; puneetsingh_bio@y7mail.com (P.S.); 19056106@student.westernsydney.edu.au (V.A.A.S.K.T.); 4 South Western Clinical School, University of New South Wales, Liverpool, NSW 2145, Australia; 5 Liverpool Clinical School, University of Western Sydney, Liverpool, NSW 2145, Australia

**Keywords:** microsatellite instability, MSI, mismatch repair deficiency, MMRD, Lynch syndrome, capillary electrophoresis, immunohistochemistry

## Abstract

Background: Microsatellite instability (MSI) testing is important for the classification of Lynch syndrome, as a prognostic marker and as a guide for adjuvant chemotherapy in colorectal cancer (CRC). The gold standard for determining MSI status has traditionally been fluorescent multiplex polymerase chain reaction (PCR) and capillary gel electrophoresis (CGE). However, its use in the clinical setting has diminished and has been replaced by immunohistochemical (IHC) detection of loss of mismatch repair protein expression due to practicability and cost. The aim of this study was to develop a simple, cost-effective and accurate MSI assay based on CGE. Method: After amplification of microsatellites by polymerase chain reaction (PCR) using the National Cancer Institute (NCI) panel (BAT 25, BAT26, D5S346, D2S123, D17S250) of MSI markers, parallel CGE was utilized to classify colorectal cancers as MSI-H, MSI-L and MSS using the 5200 Fragment Analyzer System. Cell lines and patient cancer specimens were tested. DNA from 56 formalin-fixed paraffin-embedded cancer specimens and matched normal tissue were extracted and CGE was performed. An automated computational algorithm for MSI status determination was also developed. Results: Using the fragment analyser, MSI status was found to be 100% concordant with the known MSI status of cell lines and was 86% and 87% concordant with immunohistochemistry (IHC) from patient cancer specimens using traditional assessment and our MSI scoring system, respectively, for MSI determination. The misclassification rate was mainly attributed to IHC, with only one (1.8%) sampling error attributed to CGE testing. CGE was also able to distinguish MSI-L from MSI-H and MSS, which is not possible with IHC. An MSI score based on total allelic variability that can accurately determine MSI status was also successfully developed. A significant reduction in cost compared with traditional fluorescent multiplex PCR and CGE was achieved with this technique. Conclusions: A simple, cost-effective and reliable method of determining MSI status and an MSI scoring system based on an automatic computational algorithm to determine MSI status, as well as degree of allelic instability in colorectal cancer, has been developed using the 5200 Fragment Analyzer System.

## 1. Introduction

Microsatellite instability (MSI) testing for colorectal cancer has become universal in many countries worldwide. The main utility of MSI testing has been to aid with the diagnosis of Lynch syndrome [1]. In 2016, the National Institute for Health and Care Excellence (NICE) called for universal MSI testing to guide further testing for Lynch syndrome for people with colorectal cancer [2]. Microsatellite instability status has also been used to guide adjuvant therapy [3,4,5], although the use of MSI status to guide adjuvant treatment for colorectal cancer remains controversial [6,7]. Arguably, MSI tumours may not respond as well to 5-fluorouracil (5-FU) based chemotherapy [3,8,9], but may respond better to irinotecan [10] or oxaliplatin therapy [11]. Furthermore, the European Society for Medical Oncology (ESMO) guidelines suggest that patients with Stage II colon cancer with high-risk adverse features in the absence of MSI may benefit from chemotherapy [12,13]. Several meta-analyses have shown that MSI status may be useful in guiding prognosis [14,15,16] as well as predicting the risk of dissemination [16,17]. Furthermore, MSI status alongside stage and other clinically relevant biomarkers may be used to provide patients with a more accurate prognosis as well as guide the optimal surveillance regimen post cancer resection. Finally, in the era of immunotherapy, MSI status has become an important biomarker to guide the selection of colorectal cancer patients suitable for immunotherapy [18], with the CheckMate study by Overman et al. demonstrating a durable response and disease control with Nivolumab in patients with metastatic MSI-H colorectal cancer [19].

Since 1997, an international consensus has supported the use of the Bethesda panel of five markers to detect MSI [20]. This panel of markers consists of two mononucleotide repeats BAT-25 and BAT-26 and three dinucleotides D5S346, D2S123 and D17S250. The test requires DNA extraction, PCR and resolution of the amplification products by capillary gel electrophoresis (CGE). It is commonly performed using costly fluorescent primer sets and the PCR products separated using an expensive genetic analyser instrument. Colorectal cancers with high-level microsatellite instability (MSI-H) have insertion/deletion mutations in repeats of short non-coding microsatellites (1–6 bp) [21], and as such, are characterised by alterations in nucleotide length in DNA sequences. Capillary gel electrophoresis can detect alterations in nucleotide length in DNA sequences as this test separates PCR products of different sizes, thereby enabling the characterisation of instability at different microsatellite loci. Tumours with instability at two or more of the markers are considered to be MSI-H; at one marker, they are considered to be microsatellite instability low (MSI-L) and those without instability are considered to be microsatellite stable (MSS).

While still regarded as the gold standard, molecular-based determination of MSI has fallen out of favour for clinical utilisation, mainly due to assay workflow and instrument expense. In 2009, Palomaki et al. performed an evidence review for the Evaluation of Genomic Applications in Practice and Prevention (EGAPP) working group (EQG), reviewing the cost of MSI testing by both CGE (which traditionally requires expensive fluorescent primer sets) and by immunohistochemistry (IHC) for mismatch repair deficiency (MMRD) and determined the cost to be USD 457 and USD 261, respectively [22]. While the economics of these tests have changed with time, MSI status determination for colorectal cancer for clinical purposes is still largely performed using IHC in most institutions.

**Figure 1:**
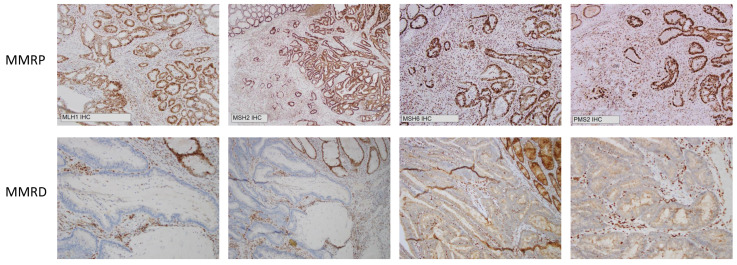
Immunohistochemistry demonstrating mismatch repair proficiency (MMRP) and mismatch repair deficiency (MMRD) at MLH1, MSH2, MSH6 and PMS2.

It is evident that IHC has worked well as a surrogate measure for determining MSI status and has been the workhorse for evaluating MSI status on a clinical basis in the era of universal MSI testing. It is practicable, cost-effective and can be applied widely [23]. However, it does not directly identify changes in DNA, but rather identifies loss of mismatch repair protein expression, specifically MLH1, MSH2, MSH6 and PMS2 (Figure 1). One limitation of IHC is that it can only classify colorectal cancer as either MSI-H or MSS. It cannot determine MSI-L, nor can it assess the degree of allelic instability. Further, approximately 5% of MSI-H tumours have normal levels of mismatch repair (MMR) proteins and are potentially be missed by IHC (these MMR proteins have retained expression but are functionally defective). Therefore, this 5% of MSI-H tumours would be misclassified as MSS [24]. A study by Cheah et al. estimated the accuracy of IHC to be 89–95% [25], whereas fluorescent multiplex PCR and CGE can achieve up to 100% accuracy [26].

Several studies have already reported different CGE based assays for MSI testing (Suraweera et al. (2002) [27], Shemirani et al. (2011) [28], Murphy et al. (2006) [29], Goel et al. (2009) [30] and Buhard et al. (2004) [31], but to the best of our knowledge, this is the first study using the Fragment Analyzer System to determine MSI status in colorectal cancer.

The primary objective of this study was to develop a molecular MSI test based on CGE (which is still considered a gold standard) [23] that is cost-effective, practicable, with rapid reporting of results; preferably with a computational algorithm that can accurately determine MSI status automatically based on the NCI panel of markers without the need for a technician/scientist/medical professional to inspect the electropherogram or digital gels to determine MSI status. The secondary objective of this study was to develop an MSI score based on allelic variability (which represents the degree of alterations in nucleotide lengths within the microsatellites) and to use this score as an alternative computational method to determine MSI status, as well as to use it as a means to further characterise MSI-H colorectal cancers.

## 2. Materials and Methods

### 2.1. Cell Lines and Culture

Twelve colorectal cancer cell lines including RKO, LS174T, HT116, LIM1215, LIM2033, LISP1, DLD1 and LOVO (representing MSI-H cell lines) and SW1222, HT29, SW620 and SW480 (representing MSS cell lines) were grown in an RPMI medium containing 10% foetal bovine serum (FBS; Gibco) and 2 mM L-glutamine (Gibco) at 37 °C and 5% CO_2_. All cell lines were harvested at 80–90% confluence using 0.05% Trypsin-EDTA (Gibco) and genomic DNA was extracted from the cell pellets for use as MSI-H and MSS controls.

### 2.2. Patient Samples

Seventy-two colorectal cancer patients with available BRAF and MSI status information (see Table 1) were identified from the Concord Colorectal Cancer Resection Database (Institutional Ethics approval: Sydney Local Health District Ethics CH62/62011-136 HREC/11/CRGH206). All patients included in this study provided written consent for the use of their information for research. Formalin-fixed paraffin-embedded (FFPE) tumour and matched normal tissue samples comprising 19 MSI-H and BRAF mutant, 23 MSI-H and BRAF wild type and 30 MSS specimens were retrieved from the Concord anatomical pathology department. Nineteen matched samples were randomly selected from each subgroup (a total of 57 samples). Routine pathologic review of all samples was performed by an expert pathologist, and after retrieval, tissue sufficiency was reviewed by JWTT and KJS. For one of the MSI-H and BRAF mutant samples, insufficient tumour material was available for DNA extraction and was excluded from the study. The remaining 56 tumour blocks were randomly assigned a study identification number (ID) and the investigators were then blinded to MSI status and patient demographics associated with each sample.

### 2.3. Immunohistochemistry Analysis

Immunohistochemical analysis for MMR protein (MLH1, MSH2, MSH6 and PMS2) expression was routinely performed on 4 µm FFPE tumour sections and stained using an automated IHC Stainer. An experienced pathologist reviewed the IHC results and confirmed MMR protein expression status for all samples used in this study, with the absence of staining within tumour regions indicating loss of MMR protein expression (MMRD).

### 2.4. DNA Extraction and Quantitation

Genomic DNA was extracted from all cell lines using the Isolate II Genomic DNA Kit (Bioline, London, UK) in accordance with the manufacturer’s instructions. After elution, the DNA was quantified using a NanoDrop spectrophotometer (Thermo Fisher Scientific, Waltham, MA, USA). For the tissue samples, five 10µm scrolls were taken from the FFPE blocks and DNA extraction was performed using the QIAamp DNA FFPE Tissue Kit (Qiagen, Hilden, Germany) according to the manufacturer’s instructions. All tissue DNA samples were quantified as per the cell line DNA.

### 2.5. PCR Amplification and Capillary Electrophoresis Detection of MSI

**Figure 2:**
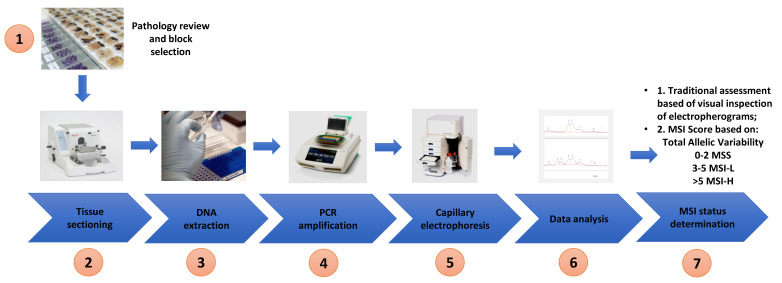
Workflow of study—pathology review and tumour block selection, tissue sectioning into scrolls, DNA extraction, polymerase chain reaction, capillary electrophoresis, inspection of electropherogram/data analysis and determination of MSI status based on allelic variability and MSI score.

Extracted DNA from both cell lines and tissue was used for MSI analysis. The optimised Bethesda panel of 5 microsatellite markers (BAT 25, BAT 26, D2S123, D5S346 and D17S250) described by Umetani et al. was used in this study and the primer sequences are shown in Table 2 [32]. PCR amplification was performed using a Bio-Rad C1000 Touch Thermal Cycler (Bio-Rad Laboratories) in 10 µL reaction mixtures containing 20 ng of genomic DNA, 0.4 µM of each primer and 1× MyTaq Mix following the recommendations of the manufacture (Bioline). The following PCR cycling conditions were used: 3 min initial denaturation at 95 °C and 40 cycles at 95 °C for 30 s, 56 °C for 30 s and 72 °C for 20 s and a final extension at 72 °C for 2 min. The PCR products were then subjected to parallel CGE using a 1–500 bp (DNF-905) DNA kit on a 5200 Fragment Analyzer System (Agilent) according to the manufactures instructions (see Figure 2 for the workflow).

### 2.6. Microsatellite Analysis

Analysis of microsatellite instability was performed by the following two approaches. Firstly, electropherograms of all samples were visually inspected and MSI status was based on the observed number of markers that displayed instability. If two or more markers were unstable, then the sample was classified as MSI-H; if there was no instability in any of the markers, then the sample was classified as MSS. If instability was found in one marker, the sample was considered to be MSI-L. Secondly, a computational algorithm based on the RFU signals assigned to tumour and normal tissue electropherogram fragment peaks was developed to automatically detect differences in DNA products without visual inspection of the electropherogram and accurately call samples as MSI-H, MSI-L or MSS. In order to achieve this, for each peak size at each position, the proportion of the signal (RFU) assigned to that position and whether the peak was ‘novel’ in the sample compared to the matched sample control. A peak was deemed ‘novel’ if it was at least 2 bases from the nearest peak in the matched normal, not more than 10 bases outside of the entire range of peak sizes observed in the normal, and if it accounted for more than 2% of the total signal in the sample (peaks with less than 2% were filtered as potential noise). This noise level may be easily adjusted in the computational method as required depending on the calibration values at each laboratory. The sum of the proportion of the signal that was assigned to novel peaks for each position for each tumour sample is then reported.

For each marker, a percentage allelic variability was calculated by this computational method. An allelic variability of >2% for any marker was considered unstable, ≤2% was considered stable. Using the traditional assessment of the NCI panel of markers, if none of the markers were unstable, the tumour was considered MSS; if one marker was unstable, MSI-L and if two or more markers were unstable, then the tumour was considered MSI-H. The maximum allelic variability of any marker (%) for each sample was assessed and an MSI score based on total allelic variability was created. An MSI score of 1–2 was considered MSS; 3–5 MSI-L; ≥5 MSI-H. A Total high-level MSI (Toh) score (/500) was attributed to each MSI-H colorectal cancer. Finally, the MSI status calculated by visual assessment of NCI markers, automatically by % allelic variability (≤2%/>2%) and by MSI score based on total allelic variability was compared to the MSI status reported by pathologist-based assessment of IHC.

### 2.7. Statistical Analysis

Statistical analysis was performed using STATA (Stata MP, Version 15; StataCorp LP) and GraphPad Prism.

## 3. Results

### 3.1. MSI Assessment of Colorectal Cancer Cell Lines

The microsatellite status of twelve colorectal cancer cell lines was analysed by CGE using the Fragment Analyzer System and the electropherograms were visually inspected (JWTT and KJS). There was 100% sensitivity and specificity in the comparative analysis of MSI status determined by CGE and the known MSI status of the cancer cell line (Table 3).

### 3.2. Limit of Detection of MSI

In order to determine the potential lower limit of tumour cellularity that MSI is able to be accurately detected in a specimen by Fragment Analyser-based CGE, a cell line mixing experiment was performed. RKO (MSI-H cell line) genomic DNA was proportionally mixed with HT-29 (MSS cell line) to 5%, 10%, 20%, 40%, 60%, 80% and 100% in a total of 20 ng of genomic material. Using allelic variability scoring of >2%/≤2% for each marker, MSI was detected even at 5% of MSI-H genomic material (Table 4). This suggested that CGE using the Fragment Analyser can detect MSI at very low concentrations and laborious micro-dissection of tumour tissue from each block may not be required.

### 3.3. Patient Tumour Specimens

Of the 72 patient specimens with known MSI and BRAF status (MSS *n* = 30, MSI-H:BRAF mutant *n* = 19 and MSI-H:BRAF wild type *n* = 23), 19 patients from each subgroup were selected for analysis and of these 57 patients, 56 patient tumour specimens were analysed by CGE.

**Figure 3:**
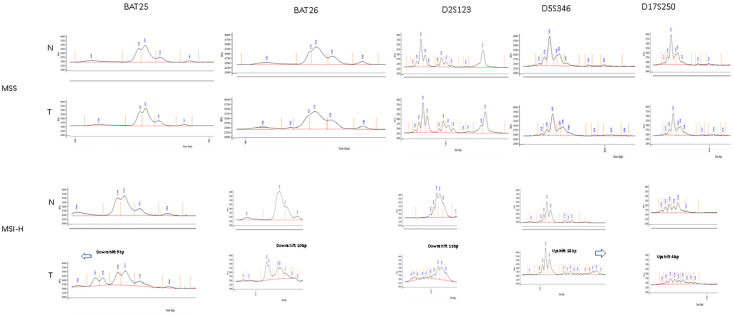
Example of visual inspection of electropherogram for tumour specimens—above (MSS) showing identical electropherograms between tumour and matched normal tissue, below (MSI-H) showing allelic shift between tumour and matched normal tissue on electropherogram.

**Figure 4:**
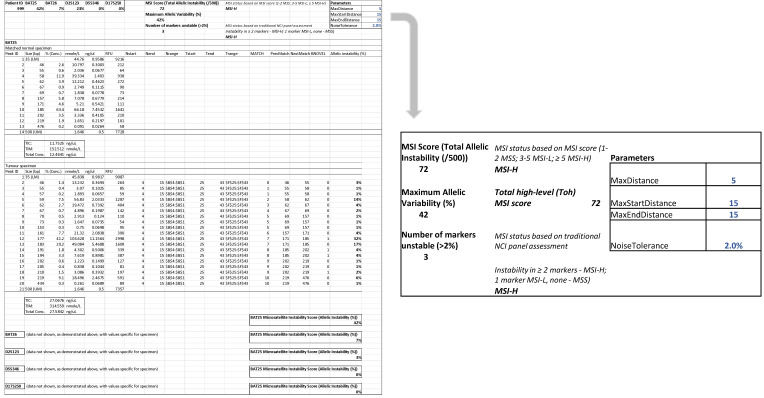
Automatic computation methods for MSI status determination based on allelic instability using both a traditional assessment of NCI panel (0/1/≥2 markers unstable corresponding to MSS/MSI-L and MSI-H respectively) and an MSI scoring system (total allelic variability 1–2/3–5/>5 corresponding to MSS/MSI-L and MSI-H). This tumour specimen was considered MSI-H with a Total high-level (Toh) MSI score of 72.

The tumour specimens were classified as MSS, MSI-L or MSI-H by assessment of the NCI panel of markers based on visual inspection of the digital gel view or electropherogram (Figure 3) and by using the automatic computational method developed in this study (Figure 4). There was 100% concordance with visual inspection and allelic variability scoring in determining if any one of the five markers displayed instability.

As IHC cannot determine MSI-L, these were excluded from correlation analysis with IHC. There was a 93% (14/15), 85% (11/13) and 79% (11/14) correlation with IHC based on an assessment of the NCI panel of markers using the allelic variability score of >2%/≤2% for each marker for MSI-H:BRAF mutant, MSI-H:BRAF wild type and MSS subgroups, respectively. In total, for the assessment of the NCI panel of markers by both computational methods of allelic variability and by visual inspection the correlation with IHC was 86%.

We also assessed MSI status using an MSI score based on total allelic variability (/500). An MSI score of 1–2 was considered MSS; 3–5 MSI-L; >5 MSI-H. Again, MSI-L was excluded from correlation analysis with IHC. Based on the MSI score, there was a 94% (17/18), 87% (13/15) and 77% (10/13) correlation with IHC. In total, the correlation between MSI score with IHC was 87%.

The correlation between CGE and IHC determination of MSI status is shown in Table 5, Table 6 and Table 7. The original MSI status (determined by IHC) on the anatomical pathology report was used for comparison and representative IHC staining for MMRP and MMRD is shown in Figure 1.

**Figure 5:**
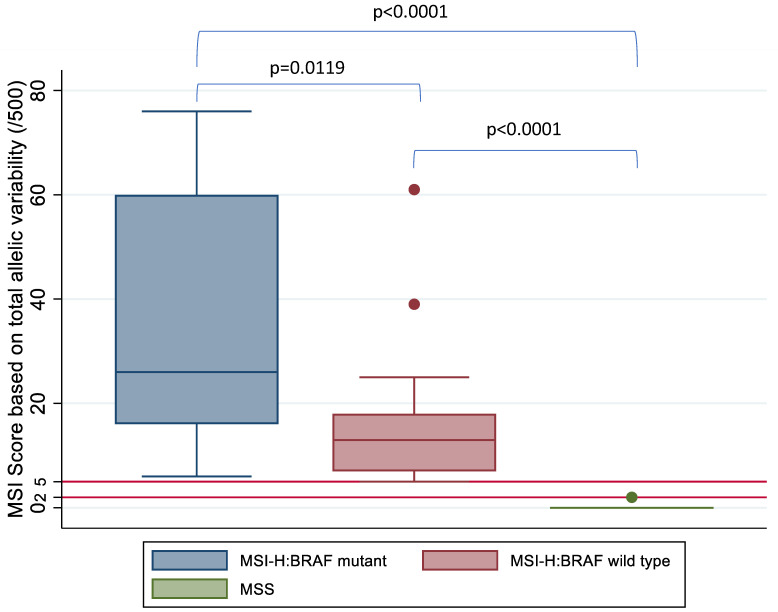
MSI score based on allelic instability (/500) by MSI and BRAF status confirmed on IHC and high-resolution capillary electrophoresis. Kruskal–Wallis test shows a statistically significant difference between the three subgroups based on MSI status. Horizontal red lines mark the tiers between MSS, MSI-L and MSI-H based on MSI score.

A Student *t*-test and Kruskal–Wallis test was used to compare the mean and median MSI score between the three groups based on MSI and BRAF status, respectively, and this demonstrated a statistically significant difference in MSI score between the MSI-H:BRAF mutant, MSI-H:BRAF wild type and MSS subgroups (Figure 5).

### 3.4. Analysis of Discordant IHC and DNA Based MSI Status

Where the MSI status reported by IHC and CGE were different, both were re-examined. There were several misclassifications by IHC. Three MSI-H colorectal cancers identified by CGE were classified as MSS by IHC. One MSI-H:BRAF mutant and two MSI-H:BRAF wild-type colorectal cancers identified by IHC were classified as MSS using CGE. There was one sampling error (re-examination of the tumour block and the H&E section revealed <5% tumour in the specimen where the MSI-H colorectal cancer was incorrectly classified as MSS on CGE). In total, compared to IHC, which had a misclassification rate of 8.9% (5/56), there was only one misclassification due to a sampling error using CGE (1.8%) (1/56).

Furthermore, 13/56 (23%) of the specimens had only one marker displaying instability based on assessment of the NCI panel using CGE and 10/56 (18%) by MSI total allelic variability score of 3–5/500. These cancer specimens were considered MSI-L by CGE, but IHC classification was not able to distinguish MSI-L from MSS and MSI-H.

## 4. Discussion

Approximately 15% of colorectal cancers are microsatellite unstable [14,15,16,33], 3% are associated with Lynch syndrome and 12% due to other causes, including epigenetic silencing of MLH1. Colorectal cancer is the third most common cancer with >1.9 million cases (representing 1 in 10 cancers) worldwide and the second most common cause of cancer death (9.4% of cancer deaths) [34]. With IHC, there may be a 5–11% misclassification rate of MSI status in colorectal cancer [24,25]. This means that approximately 95,000–209,000 colorectal cancers are incorrectly classified into the wrong MSI subgroup. Furthermore, IHC fails to distinguish MSI-L from MSI-H and MSS, as IHC only classifies cancers into MSI-H/MSS. Thus, the use of only one detection method alone may lead to misdiagnosis of mismatch repair deficiency status in a small number of cases [35]. This is because IHC examines protein expression rather than DNA microsatellite sequences. In cases where MMR proteins have retained expression despite being functionally defective, IHC cannot detect MMRD.

However, most institutions perform IHC due to the significant expense and practicability issues with the complex techniques associated with DNA based testing [22], despite CGE being the gold standard [23]. With this in mind, the aim of our study was to develop a simple, accurate and cost-effective MSI test using CGE and utilizing (i) basic assessment and (ii) development of an MSI score to represent the total allelic variability of the tumour.

Currently, several different MSI analysis systems based on CGE are being used [26,27,28,29,30,31]. The strength of the MSI assay we have developed using the Fragment Analyzer System is that it is accurate, simple, practicable, automated and cost-effective. In this study, a sensitivity and specificity of 100% in determining MSI status in cancer cell lines was observed. This was similar to the study by Arulananda et al., which also reported 100% accuracy with fluorescent multiplex PCR and CGE [26]. On patient specimens, when using a basic assessment of the NCI panel of markers using both automatic computational methods and visual inspection of the electropherogram, the correlation with IHC was 86%. When using the MSI score, the correlation with IHC was 87%. This is in line with other IHC studies, with the reported accuracy of IHC being 89–95% [25]. There was only one error attributed to CGE, and this was noted to be an error with tumour sampling rather than an error with the testing regimen. CGE was able to detect allelic variability within 2 bp for fragments between 1–500 bp [36].

In terms of practicability, micro-dissection prior to DNA extraction was not required. With the 5200 Fragment Analyzer System, up to 24 cancer specimens may be tested per run (10 wells/1 row for each specimen (tumour and matched normal for each of the five markers and one well for the ladder) as each tray has 96 wells (8 rows × 12 columns) and three trays may be accommodated for each run). Furthermore, an accurate computational method to determine MSI status without visual inspection of the electropherogram has been developed in this study, requiring less user intervention and ensuring rapid reporting.

The cost of the assay developed in this study was approximately $25, compared to IHC, which costs > $100. We have thus achieved a significant cost reduction. Unlike traditional fluorescent multiplex PCR and CGE, expensive fluorescent labelled primers were not required with this test.

While techniques other than IHC and CGE for MSI status determination exist, including NGS techniques (which targets known genes for genome sequencing) [37], single-molecule molecular inversion probes (snMIPs) (which do not need matched normal tissue) [38] and the MANTIS calculation method (which requires comparisons with tumour cell detection and stained histological section images to predict MSI status) [39], these techniques have their own limitations and disadvantages [23].

Prior to this study, the issues with using CGE or using two methods of MSI detection (IHC and CGE) were its high detection cost and high sample demand [35]. In this study, we have achieved an accurate, cost-effective CGE-based test capable of high throughput with ease of use and automatic computational methods for MSI status determination for colorectal cancer. While MSI is most commonly associated with colorectal cancer, MSI may also be found in gastric, endometrial, ovarian, urinary tract, hepatobiliary, brain and skin cancers. The CGE technique and automatic computational methods developed in this study may also be useful in the determination of MSI status in these other cancers.

### Limitations

In most studies using CGE, tumour tissue is micro-dissected prior to DNA extraction [29]. In this study, micro-dissection of the tumour was not performed to keep the technique simple and practicable. Instead, H&E slides were microscopically examined and specimen blocks with greater than 10% tumour were used for DNA extraction. However, in this study, one tumour block with <5% tumour tissue was utilized, leading to a sampling error. While not performing micro-dissection may lead to errors if there is insufficient tumour in the block, this study showed in the mixing experiment with cell lines that the MSI status of samples with ≥5% MSI genomic material can be accurately classified even at low tumour content.

While this test was able to accurately determine allelic instability, the clinical significance of allelic instability remains unclear. It has been shown that tumour mutational burden is an emerging biomarker for response to checkpoint inhibitors [40], but it is unknown if allelic instability may also be a biomarker for response to checkpoint inhibition and this may be an area of interest in future research direction.

## 5. Conclusions

A simple, cost-effective and accurate test based on CGE using the Fragment Analyzer System linked with an automated computational method to call MSI status without the need for visual inspection of the electropherogram has been developed and potentially useful in both research and clinical settings. An MSI score based on total allelic instability, which can accurately determine MSI status, as well as correlate with the degree of genetic instability of the cancer, has also been developed in this study. Future research directions may include evaluating if this MSI score correlates with tumour mutational burden and response to checkpoint inhibitors.

## Figures and Tables

**Table 1:** Patient and tumour characteristics based on MSI and BRAF status.

	MSS	%	MSI-H BRAF Mutant	%	MSI-H BRAF Normal	%
n	30		19		23	
F	5	17%	9	47%	12	52%
M	25	83%	10	53%	11	48%
ASA (mean, SD)	2.2, 0.9		3, 1		2, 1	
Age (mean, SD)	68, 14		80, 7		67, 16	
Age (median)	66		81		66	
Liver metastases	3	10%	2	11%	2	9%
Lung metastases	3	10%	2	11%	2	9%
Brain metastases	3	10%	2	11%	2	9%
Nodal metastases	3	10%	2	11%	2	9%
Stage I	10	33%	2	11%	8	35%
Stage II	12	40%	10	53%	9	39%
Stage III	5	17%	6	32%	4	17%
Stage IV	3	10%	1	5%	2	9%
Caecum	2	7%	4	21%	2	9%
Ascending Colon	1	3%	1	5%	5	22%
Hepatic Flexure	0	0%	1	5%	1	4%
Transverse Colon	4	13%	6	32%	3	13%
Splenic Flexure	1	3%	0	0%	1	4%
Descending Colon	0	0%	2	11%	1	4%
Sigmoid Colon	12	40%	3	16%	1	4%
Rectum	10	33%	2	11%	9	39%
Total Right	7	23%	12	63%	11	48%
Total Left	23	77%	7	37%	12	52%
TIL present	1	3%	12	63%	8	35%
TIL inconspicuous	29	97%	7	37%	15	65%
Tumour size (mean, cm)	4.6		4.6		4.4	
Low grade	1	3%	0	0%	0	0%
Moderate grade	26	87%	13	68%	18	78%
High grade	3	10%	6	32%	5	22%
Poorly differentiated	3	10%	5	26%	5	22%

**Table 2:** Bethesda panel primer sequences for microsatellite analysis.

Name	Primer Sequence 5′ to 3′	TM (°C)
BAT-25	5′-TCGCCTCCAAGAATGTAAGT-3′ (F)	57.1
	5′-TCTGCATTTTAACTATGGCTC-3′ (R)	54.5
BAT-26	5′-TGACTACTTTTGACTTCAGCC-3′ (F)	54.4
	5′-AACCATTCAACATTTTTAACCC-3′ (R)	56.8
D5S346	5′-TACTCACTCTAGTGATAAATCGG-3 (F)	56.3
	5′-TTCAGGGAATTGAGAGTTACAG-3′ (R)	52.2
D2S123	5′-GCCAGAGAAATTAGACACAGTG-3′ (F)	52.8
	5′-CTGACTTGGATACCATCTATCTA-3′ (R)	55.8
D17S250	5′-AATAGACAATAAAAATATGTGTGTG-3′ (F)	52
	5′-TATATATTTAAACCATTTGAAAGTG-3′ (R)	51.7

**Table 3:** Sensitivity, specificity, positive and negative predictive value of high-resolution capillary electrophoresis of colorectal cancer cell lines by visual inspection of high-resolution capillary electrophoresis signatures.

Colorectal Cancer Cell Lines	Expected MSI Status	Experimental MSI Status		
SW480	MSS	MSS	Sensitivity	100%
SW620	MSS	MSS	Specificity	100%
SW1222	MSS	MSS	PPV	100%
HT29	MSS	MSS	NPV	100%
LS174T	MSI-H	MSI-H		
RKO	MSI-H	MSI-H		
LISP1	MSI-H	MSI-H		
DLD1	MSI-H	MSI-H		
LOVO	MSI-H	MSI-H		
HT116	MSI-H	MSI-H		
LIM1215	MSI-H	MSI-H		
LIM2033	MSI-H	MSI-H		

**Table 4:** Limit of microsatellite instability detection analysis in cell lines. MSI dilution series (5%, 10%, 20%, 40%, 60%, 80%, 100%) using RKO (MSI-H) and HT29 (MSS) cell line DNA.

ID	MSI Status Based on IHC	BAT-25	BAT-26	D5S346	D2S123	D17S250	^†^ No. of NCI (/5) Markers >2% Instability in ≥2 Markers: MSI-H; 1 Marker MSI-L; None: MSS	MSI Status	Maximum Allelic Variability (/100)	MSI Score Based on Total Allelic Variability (/500)	^‡^ MSI Status Based on Total Allelic Variability (MSI Score = 1–2 MSS; 3–5 MSI-L; ≥5 MSI-H)
100% RKO	MSI-H 100%	7%	0%	36%	0%	13%	3	MSI-H	36%	56	MSI-H
80% RKO	MSI-H 80%	42%	7%	23%	0%	0%	3	MSI-H	42%	73	MSI-H
60% RKO	MSI-H 60%	11%	0%	21%	6%	8%	4	MSI-H	21%	46	MSI-H
40% RKO	MSI-H 40%	11%	0%	18%	0%	22%	3	MSI-H	22%	51	MSI-H
20% RKO	MSI-H 20%	6%	0%	17%	0%	35%	3	MSI-H	35%	58	MSI-H
10% RKO	MSI-H 10%	0%	5%	3%	0%	17%	3	MSI-H	17%	25	MSI-H
5% RKO	MSI-H 5%	10%	0%	0%	0%	18%	2	MSI-H	18%	29	MSI-H

^†^ NCI panel–Traditional definition of MSI status based on number of markers demonstrating instability (100% MSI-H detection). ^‡^ NCI panel–MSI score based on total allelic variability demonstrating instability (100% MSI-H detection).

**Table 5:** Correlation between MSI status detection using high-resolution capillary electrophoresis with (a) traditional analysis and (b) MSI score based on total allelic variability, compared to immunohistochemistry (IHC) for MSI-H BRAF mutant tumours from patient specimens.

ID	MSI Status Based on IHC	BAT-25	BAT-26	D5S346	D2S123	D17S250	^†^ No. of NCI (/5) Markers >2% Instability in ≥2 Markers: MSI-H; 1 Marker MSI-L; None: MSS	MSI Status	Maximum Allelic Variability (/100)	^‡^ MSI Score Based on Total Allelic Variability (/500)	MSI Status Based on Total Allelic Variability (MSI Score = 1–2 MSS; 3–5 MSI-L; ≥5 MSI-H)
ID 14	MSI-H BRAF mutant	4%	14%	0%	10%	3%	4	MSI-H	14%	31	MSI-H
ID 24	MSI-H BRAF mutant	24%	36%	0%	0%	0%	2	MSI-H	36%	61	MSI-H
ID 25	MSI-H BRAF mutant	7%	20%	0%	0%	0%	2	MSI-H	20%	26	MSI-H
ID 29	MSI-H BRAF mutant	2%	4%	0%	0%	0%	1	MSI-L *	4%	6	MSI-H
ID 31	MSI-H BRAF mutant	16%	41%	0%	0%	3%	3	MSI-H	41%	60	MSI-H
ID 32	MSI-H BRAF mutant	5%	0%	3%	0%	0%	2	MSI-H	5%	8	MSI-H
ID 34	MSI-H BRAF mutant	0%	0%	0%	0%	0%	0	MSS	0%	0	MSS
ID 35	MSI-H BRAF mutant	11%	0%	9%	0%	0%	2	MSI-H	11%	20	MSI-H
ID 38	MSI-H BRAF mutant	13%	34%	12%	0%	0%	3	MSI-H	34%	59	MSI-H
ID 42	MSI-H BRAF mutant	16%	28%	9%	5%	0%	4	MSI-H	28%	58	MSI-H
ID 47	MSI-H BRAF mutant	25%	38%	9%	0%	4%	4	MSI-H	38%	76	MSI-H
ID 52	MSI-H BRAF mutant	0%	11%	0%	0%	5%	2	MSI-H	11%	16	MSI-H
ID 55	MSI-H BRAF mutant	7%	54%	3%	0%	8%	4	MSI-H	54%	71	MSI-H
ID 45	MSI-H BRAF mutant	0%	5%	0%	2%	0%	1	MSI-L *	5%	7	MSI-H
ID 142	MSI-H BRAF mutant	3%	7%	5%	0%	7%	3	MSI-H	7%	21	MSI-H
ID 252	MSI-H BRAF mutant	0%	8%	7%	0%	0%	2	MSI-H	8%	14	MSI-H
ID 422	MSI-H BRAF mutant	0%	0%	0%	67%	0%	1	MSI-L *	67%	67	MSI-H
ID 242	MSI-H BRAF mutant	2%	12%	6%	0%	0%	3	MSI-H	12%	20	MSI-H

* Tumour samples considered MSI-L based on NCI panel were excluded from correlation with IHC analysis as IHC analysis cannot determine MSI-L status. ^†^ 93% correlation with IHC. ^‡^ 94% correlation with IHC.

**Table 6:** Correlation between MSI status detection using high-resolution capillary electrophoresis with (a) traditional analysis and (b) MSI score based on total allelic variability, compared to immunohistochemistry (IHC) for MSI-H:BRAF wild type tumours from patient specimens.

ID	MSI Status Based on IHC	BAT-25	BAT-26	D5S346	D2S123	D17S250	^†^ No. of NCI (/5) Markers >2% Instability in ≥2 Markers: MSI-H; 1 Marker MSI-L; None: MSS	MSI Status	Maximum Allelic Variability (/100)	^‡^ MSI Score Based on Total Allelic Variability (/500)	MSI Status Based on Total Allelic Variability (MSI Score = 1–2 MSS; 3–5 MSI-L; >5 MSI-H)
ID 1	MSI-H BRAF wild type	12%	5%	4%	0%	5%	4	MSI-H	12%	25	MSI-H
ID 2	MSI-H BRAF wild type	5%	6%	0%	0%	2%	2	MSI-H	6%	13	MSI-H
ID 8	MSI-H BRAF wild type	24%	22%	9%	0%	6%	4	MSI-H	24%	61	MSI-H
ID 9	MSI-H BRAF wild type	4%	0%	0%	0%	9%	2	MSI-H	9%	13	MSI-H
ID 16	MSI-H BRAF wild type	0%	0%	0%	0%	0%	0	MSS	0%	0	MSS
ID 17	MSI-H BRAF wild type	0%	0%	0%	6%	0%	1	MSI-L *	6%	6	MSI-H
ID 18	MSI-H BRAF wild type	0%	4%	3%	0%	0%	2	MSI-H	4%	7	MSI-H
ID 27	MSI-H BRAF wild type	0%	5%	0%	0%	0%	1	MSI-L	5%	5	MSI-L
ID 40	MSI-H BRAF wild type	2%	0%	3%	0%	0%	1	MSI-L *	3%	5	MSI-L
ID 46	MSI-H BRAF wild type	4%	0%	0%	0%	4%	2	MSI-H	4%	8	MSI-H
ID 56	MSI-H BRAF wild type	3%	0%	2%	0%	0%	1	MSI-L *	3%	5	MSI-L
ID 58	MSI-H BRAF wild type	0%	5%	0%	4%	5%	3	MSI-H	5%	13	MSI-H
ID 63	MSI-H BRAF wild type	10%	2%	0%	0%	5%	2	MSI-H	10%	17	MSI-H
ID 64	MSI-H BRAF wild type	5%	26%	8%	0%	0%	3	MSI-H	26%	39	MSI-H
ID 72	MSI-H BRAF wild type	0%	0%	0%	4%	0%	1	MSI-L *	4%	4	MSI-L
ID 152	MSI-H BRAF wild type	2%	2%	8%	0%	0%	1	MSI-L *	8%	12	MSI-H
ID 172	MSI-H BRAF wild type	14%	0%	4%	0%	0%	2	MSI-H	14%	18	MSI-H
ID 232	MSI-H BRAF wild type	0%	0%	4%	0%	9%	2	MSI-H	9%	13	MSI-H
ID 272	MSI-H BRAF wild type	0%	0%	0%	0%	0%	0	MSS	0%	0	MSS

* Tumour samples considered MSI-L based on the NCI panel were excluded from correlation with IHC analysis as IHC analysis cannot determine MSI-L status. ^†^ 85% correlation with IHC. ^‡^ 87% correlation with IHC.

**Table 7:** Correlation between MSI status detection using high-resolution capillary electrophoresis with (a) traditional analysis and (b) MSI score based on total allelic variability, compared to immunohistochemistry (IHC) for MSS tumours from patient specimens.

ID	MSI Status Based on IHC	BAT-25	BAT-26	D5S346	D2S123	D17S250	^†^ No. of NCI (/5) Markers >2% Instability in ≥2 Markers: MSI-H; 1 Marker MSI-L; None: MSS	MSI Status	Maximum Allelic Variability (/100)	^‡^ MSI Score Based on Total Allelic Variability (/500)	MSI Status Based on Total Allelic Variability (MSI Score = 1–2 MSS; 3–5 MSI-L; >5 MSI-H)
ID 3	MSS	8%	11%	0%	4%	0%	3	MSI-H	11%	24	MSI-H
ID 4	MSS	0%	0%	0%	0%	0%	0	MSS	0%	0	MSS
ID 5	MSS	0%	0%	0%	0%	0%	0	MSS	0%	0	MSS
ID 11	MSS	0%	0%	0%	0%	0%	0	MSS	0%	0	MSS
ID 15	MSS	0%	2%	0%	0%	0%	0	MSS	2%	2	MSS
ID 19	MSS	4%	0%	0%	0%	0%	1	MSI-L *	4%	4	MSI-L
ID 21	MSS	0%	0%	0%	0%	5%	1	MSI-L *	5%	5	MSI-L
ID 28	MSS	1%	0%	0%	1%	1%	0	MSS	1%	4	MSI-L
ID 33	MSS	0%	0%	0%	0%	0%	0	MSS	0%	0	MSS
ID 48	MSS	0%	0%	0%	0%	3%	1	MSI-L *	3%	3	MSI-L
ID 50	MSS	0%	0%	4%	0%	0%	1	MSI-L *	4%	4	MSI-L
ID 61	MSS	0%	3%	0%	0%	0%	1	MSI-L *	3%	3	MSI-L
ID 65	MSS	0%	0%	0%	0%	0%	0	MSS	0%	0	MSS
ID 67	MSS	15%	10%	0%	0%	4%	3	MSI-H	15%	28	MSI-H
ID 69	MSS	6%	0%	0%	0%	4%	2	MSI-H	6%	9	MSI-H
ID 70	MSS	0%	2%	0%	0%	0%	0	MSS	2%	2	MSS
ID 71	MSS	0%	0%	0%	0%	0%	0	MSS	0%	0	MSS
ID 522	MSS	0%	0%	0%	0%	0%	0	MSS	0%	0	MSS
ID 712	MSS	0%	0%	0%	0%	0%	0	MSS	0%	0	MSS

* Tumour samples considered MSI-L based on NCI panel were excluded from correlation with IHC analysis as IHC analysis cannot determine MSI-L status. ^†^ 79% correlation with IHC, ^‡^ 77% correlation with IHC.

## Data Availability

Data are available on request.
